# Sustained Immune Persistence Five Years Post-Completion of Four-Dose sIPV Vaccination

**DOI:** 10.3390/vaccines13030253

**Published:** 2025-02-27

**Authors:** Chu Kai, Li Yurong, Liu Sheng, Shan Yongmei, Wang Jianfeng, Li Xinge, Jiao Peng, Pan Hongxing

**Affiliations:** 1 Institute of Clinical Evaluation of Vaccines, Jiangsu Provincial Center for Disease Control and Prevention, Nanjing 210009, China; chukai19812007@163.com; 2 Sinovac Life Sciences Co., Ltd., Beijing 102629, China; liyr8134@sinovac.com; 3 Pizhou City Center for Disease Control and Prevention, Pizhou 221000, China; pzcdcjc@126.com; 4 Guanyun County Center for Disease Control and Prevention Preventive, Lianyungang 222200, China; gycdcsym@163.com; 5 Sinovac Biotech Co., Ltd., Beijing 100085, China; wangjianfeng@sinovac.com (W.J.); lixg@sinovac.com (L.X.)

**Keywords:** Sabin strain, inactivated poliovirus vaccine, immune persistence

## Abstract

Background: The previous study assessed the immune durability of the Sabin strain inactivated poliovirus vaccine (sIPV) at four years of age; an update on its long-term persistence is warranted. Methods: This Phase IV, open-label, parallel-controlled observational study, required by China’s National Medical Products Administration (NMPA), involves 6.5-year-old children who received four doses of sIPV or Salk IPV (wIPV) at 2, 3, 4, and 18 months during the Phase III trial. Participants are recruited in a 2:1 ratio and contribute blood samples for polio-neutralizing antibody (nAb) assays to determine non-inferiority of immune persistence. Results: The study enrolled 483 participants aged 6.5 years in the 5-year Immune Persistence Set (IPS2), with 318 in the sIPV group and 165 in the wIPV group. Additionally, 387 participants (255 sIPV, 132 wIPV) with samples at six-time points were included in the Full Sequence Immune Persistence Set (IPS3). In IPS2, seropositivity rates (SPRs) for nAbs against serotypes 1–3 were over 99% in sIPV and 98% in wIPV. At 6.5 years, geometric mean titers (GMTs) were significantly higher in the sIPV group 543.96, 179.59 and 362.72 compared to the wIPV group 190.75, 81.05 and 203.95 for serotypes 1, 2 and 3, respectively. Participants in IPS3 demonstrated comparable SPRs and GMTs to IPS2, with values of 566.01 vs. 187.41 for serotype I, 177.55 vs. 78.01 for serotype II, and 365.47 vs. 190.31 for serotype III in the sIPV and wIPV groups, respectively. From one-month post-booster to 6.5 years, nAb GMTs showed declines: 19.35-fold for serotype I, 28.12-fold for serotype II, and 32.45-fold for serotype III in the sIPV group, and 23.42-fold, 23.83-fold, and 34.54-fold in the wIPV group, respectively. Non-inferiority of nAb SPRs and GMTs for sIPV compared to wIPV among participants aged 6.5 years was confirmed for all serotypes in IPS2 and IPS3. Conclusions: The sIPV maintains good immunological persistence five years after four doses of vaccination, with nAb GMT exceeding the seroprotecting threshold, suggesting that booster doses might be currently unwarranted.

## 1. Introduction

Over the past decade, global cooperation has led to significant progress in poliomyelitis eradication. World Health Organization (WHO) announced the elimination of wild poliovirus type 2 (WPV2) in 2015 and WPV3 in 2019 [1]. In 1994, the WHO Region of the Americas was certified as polio-free. Subsequently, the WHO Western Pacific Region in 2000 and the WHO European Region in June 2002 were also certified [1,2]. However, the final step to eradication remains challenging [1].

In recent years, geopolitical conflicts, insecurity, inaccessibility, and vaccination hesitancy have significantly hindered vaccination coverage, thereby impeding the elimination of wild-type 1 poliovirus strains. In 2021, cases of wild-type 1 poliovirus were reported in Malawi and Mozambique, with the virus strains traced back to Pakistan. The discovery of wild poliovirus cases outside of Pakistan and Afghanistan, the two endemic countries, underscores the urgent need for countries to prioritize polio immunization campaigns and strengthen virus surveillance efforts [3].

The type 2 circulating vaccine-derived poliovirus (cVDPV2) poses a major public health risk akin to wild poliovirus (WPV) [4]. The 2016 shift from trivalent to bivalent oral poliovirus vaccine (tOPV to bOPV) reduced serotype 2 immunity in some areas, especially in infants born post-switch, due to declining mucosal immunity and inadequate inactivated poliovirus vaccine (IPV) coverage [3]. This immunity gap sparked widespread VDPV outbreaks, with cases soaring from 318 in 15 countries (2010–2016) to 3129 in 41 countries after OPV2 withdrawal [3,5]. Despite enhanced surveillance and vaccination rates exceeding pre-COVID-19 levels, the 2023 polio eradication goal was missed [3,6]. The Global Polio Eradication Initiative (GPEI) postponed WPV1 eradication certification to 2026 and cVDPV2 transmission cessation to 2025 [6]. As OPV use hinders global polio eradication, adopting IPV and further attenuating Sabin IPV are vital [7,8]. In 2020, the Strategic Advisory Group of Experts on Immunization (SAGE) recommended at least two IPV doses [9]. To overcome tOPV-to-bOPV switch challenges and ensure polio eradication beyond 2030, widespread IPV vaccination through additional campaigns and robust coverage is essential [1].

In developed regions, a 4-dose IPV regimen is standard, but Taiwan, Singapore, and Hong Kong use 5–6 doses. With sIPV’s rise, research is needed on long-term immune durability and IPV (including sIPV) nAb GMT trends [10,11]. Sinovac’s sIPV vaccine, proven immunogenic and safe [12,13,14,15,16], got NMPA approval in 2021 and WHO prequalification in 2022. China’s “2 + 2” strategy involves 2 IPV + 2 OPV doses with an OPV booster at age 4 [17], while some provinces and self-paid markets use a 4-dose IPV schedule. Our study, using a 4-dose schedule since Phase III [15], assessed 4-year immune persistence in Phase IV [18] and will evaluate 5-year persistence at age 6.5.

## 2. Methods

### 2.1. Study Design

This Phase IV study, designed as an open-label, parallel-controlled observational trial, builds on a previous Phase III trial (ClinicalTrials.gov Identifier: NCT03526978) and complies with the NMPA Certificate of Registration (No. 2021S00781). This Phase IV study was conducted from 2021 to March 2024 in Pizhou City and Guanyun County, China, by the Jiangsu CDC. The study aimed to enroll at least 450 participants who had completed four vaccinations, with a 2:1 ratio of sIPV to wIPV groups. It strictly adheres to the Declaration of Helsinki, good clinical practice (GCP) and all relevant ethical standards.

Participants in this study received four doses of vaccination (primary schedule of 2, 3, and 4 months with booster at 18 months) in the completed Phase III clinical trial. This study obtained informed consent separately from the Phase III clinical trial participants for blood collection conducted 2.5 years and 5 years after the completion of the four-dose vaccination series. A straightforward sampling approach was employed, targeting townships with more populations, with each township having been randomized in the Phase III trial. Recruitment proceeded until the target sample size was reached.

Each participant provided approximately 3.0 mL of blood for nAb assays to assess the non-inferiority of immune persistence among groups. Written informed consent was obtained from parents or legal guardians prior to enrollment. The trial protocol and consent form were reviewed and approved by the Ethics Committee of the Jiangsu CDC (JSJK2021-A012-01). The study is registered at ClinicalTrials.gov with the identifier NCT04989231.

### 2.2. Sample Sizes

Antibody SPRs served as the key observational metrics between the sIPV and wIPV groups, allowing for a comparative analysis of antibodies against the three serotypes. Drawing on data from prior sIPV clinical studies, it was anticipated an antibody seropositive rate (SPR) of approximately 90% for the three serotypes in the wIPV group five years post-vaccination. The sIPV-to-wIPV group sample size ratio was set at 2:1, with a significance level of α = 0.025 (one-tailed) and a power of 1 − β/3 = 1 − 0.2/3 = 93.33%. The non-inferiority margin δ was set at −10%. Utilizing NCSS-PASS 2021 software, we determined that the required sample sizes for the investigational and wIPV groups were 298 and 149, respectively. Finally, it was determined that there would be at least 300 participants in the sIPV group and 150 in the wIPV group.

### 2.3. Participants

Participants with a history of poliovirus antigen-containing vaccinations outside of the Phase III trials were excluded. IPS1 comprises all participants who completed blood collection 2.5 years post-4-dose vaccination (at the age of 4 years) with valid nAb values within the specified timeframe (ranging from 48 to 54 months of age); results from this point have been reported [18]. IPS2 includes those who completed blood collection 5 years post-4-dose vaccination (aged 6.5 years) mark with valid nAb values within the specified timeframe (ranging from 78 to 84 months).

In the Phase IV study, participant groups in IPS1 and IPS2 varied owing to separate informed consent processes at 2.5 and 5 years following the four-dose vaccination. To address this, supplementary analyses (IPS3) were conducted, focusing on the overlapping population between IPS1 and IPS2. Phase III data were then retrospectively reviewed, ensuring a consistent dataset from the same cohort, covering the period from pre-vaccination to 5 years post-vaccination, thus offering a more precise depiction of the change trajectory.

IPS3 includes the entire sequence of participants, featuring those with valid nAb values obtained both before and one month after their primary and booster immunizations, as well as at the ages of 4 years (ranging from 48 to 54 months) and 6.5 years (ranging from 78 to 84 months), corresponding to 2.5 years and 5 years post the completion of the four-dose vaccination regimen, respectively.

### 2.4. Vaccines

In the completed Phase III trials, Sinovac’s sIPV (Vero cell) was the investigational vaccine, with antigenic contents of 15 D-antigen units (DU) for type 1, 45 DU for type 2, and 45 DU per 0.5 mL dose for type 3, supplied in liquid form. The Salk strain-based control vaccine, Sanofi Pasteur’s wIPV, is produced by inactivating wild poliovirus strains (Mahoney [type 1], MEF-1 [type 2], and Saukett [type 3]) while keeping their antigenicity. Each 0.5 mL dose contains 40 DU of Mahoney strain (type 1), 8 DU of MEF-1 strain (type 2), and 32 DU of Saukett strain (type 3), all cultured on Vero cells and administered as a 0.5 mL dose.

### 2.5. Assays

Serum prepared from blood samples was stored at temperatures not exceeding −18 °C, with detailed records of its transfer, separation, and preservation processes.

To assess the immunogenicity of the vaccines, titers of serotypes 1, 2, and 3 poliovirus-specific nAbs were determined using a microneutralization test (MNT). A seropositive seroprotection status was also defined as titers of ≥1:8 [19,20].

The serum samples from the completed phase III clinical trial had been assayed by the National Institutes for Food and Drug Control (NIFDC). To maintain comparability, the serum samples of this study have also been submitted to NIFDC for nAb titer assessment. The MNT method was employed for laboratory detection using the Sabin poliovirus strain (source: WHO). The sIPV procedures adhered strictly to the guidelines outlined in the “WHO: Polio Laboratory Manual 2004” and the associated laboratory detection standard operating procedures (SOP) [21].

The serum was inactivated at 56 °C in a water bath for 30 min. Starting from a 1:4 dilution, serum was serially diluted twofold with cell maintenance medium to an appropriate dilution, with each sample being diluted in parallel in two wells. The virus was diluted to a titer of 100 CCID_50_/0.05 mL. Serum at different dilutions was mixed with an equal volume of virus solution (50 μL + 50 μL) and incubated at 35.0 °C in a 5% CO_2_ incubator for 3 h. After incubation, 100 μL of Hep-2 cell suspension (cell concentration controlled at 0.8~1.0 × 10^5^ cells/mL) was added to each well and cultured in a 35.0 °C 5% CO_2_ incubator for 7 days. Negative serum control, positive reference serum control, and normal cell controls were established, and a virus back-titration experiment was set up to detect the quantity of the virus. After 5 to 7 days of cultivation, cytopathic effects were observed, and the nAb titer of the serum samples was determined based on the results of the cytopathic observation.

### 2.6. Outcomes

The primary endpoint is the seropositivity (≥1:8) rate, that is, the seroprotection rate of nAbs, and the secondary endpoint is nAbs GMT. Non-inferiority was defined as follows: (i) the lower limit of the two-sided 95% confidence intervals (95% CI) for the SPRs difference in SPRs between the sIPV and wIPV groups must be greater than −10%, and (ii) the lower limit of the two-sided 95% CI for the GMT ratio (investigational group/wIPV group) must exceed 0.67.

### 2.7. Statistical Analysis

The statistical analyses were performed using the SAS 9.4 Software (SAS Institute Inc., Cary, NC, USA). Calculated the SPRs for the three serotypes at different time points for both the sIPV group and wIPV groups, determined their two-sided 95% CI using the Clopper-Pearson method, and statistically compared the differences between groups using the Fisher’s exact probability test; additionally, performed statistical tests on group differences (sIPV group—wIPV group) using the Fisher’s exact probability test and calculated the two-sided 95% CI for the SPRs differences for the three serotypes, conducting a non-inferiority comparison.

The GMT and two-sided 95% CI were computed for the three serotypes at various time points. The GMT ratios (sIPV group/wIPV group) and their corresponding 95% CI were also calculated. Following logarithmic transformation, a t-test was employed to statistically assess the differences between the groups. Non-inferiority was evaluated based on the two-sided 95% CI for the GMT ratios of antibodies across the three serotypes.

All statistical tests will consider a *p*-value less than 0.05 as indicating statistically significant differences.

## 3. Results

### 3.1. Study Participants

**Figure 1:**
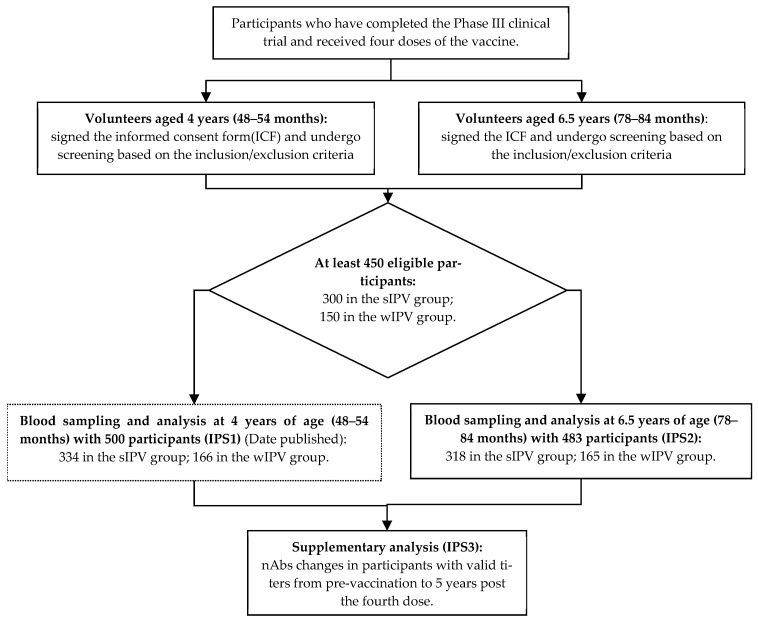
Flowchart of the Phase IV Clinical Study and Participant Analysis Dataset.

For the 5-year post-vaccination immune persistence assessment at age 6.5, 483 participants provided blood samples, with 318 in the sIPV group and 165 in the wIPV group, all with valid nAb values and included in IPS2. IPS3 included 387 participants, with 255 in the sIPV group and 132 in the wIPV group. For the detailed process of this Phase IV clinical study and the participant analysis dataset, please refer to Figure 1.

No statistically significant differences in age and gender were observed between the sIPV and wIPV groups, suggesting that the groups were balanced and comparable (see Table 1).

### 3.2. Five-Year Immune Persistence Set

Five years post-4-dose vaccination (6.5 years old), the GMTs of nAbs for serotypes I, II, and III in the sIPV group were significantly higher than those in the wIPV group, with statistically significant differences (*p* < 0.0001). Specifically, the GMT of nAbs for all serotypes in the sIPV group ranged from 179.59 to 543.96 and in the wIPV group from 81.05 to 203.95. Additionally, the SPRs for all serotypes in the sIPV group were above 99%, while in the wIPV group they were above 98%, with no statistically significant differences between the groups for serotypes 1, 2, and 3.

Further analysis of the immunogenicity for each serotype showed that the lower limits of the 95% CI for the differences in SPRs were all greater than −10%, indicating non-inferiority; the ratios of GMTs (95% CI) for each serotype were 2.85 (2.35, 3.46), 2.22 (1.86, 2.64), and 1.78 (1.44, 2.19), with the lower limits of the 95% CI all greater than 0.67, indicating non-inferiority (see Table 2).

### 3.3. Full Series Immune Persistence Set

From one month post-primary immunization to the 5 years post-4-dose vaccination (6.5 years old), at various observation time points, the nAbs GMT for serotypes I–III in the sIPV groups were significantly higher than those in the wIPV group (*p* < 0.0001). The results of IPS 3 at 5 years post-4-dose vaccination (6.5 years old) were essentially consistent with IPS 2, with both the sIPV and wIPV groups showing nAbs SPRs close to 100% (see Table 3).

At 5 years post-4-dose vaccination (6.5 years old), the nAbs GMT reduction fold of each serotype in the sIPV and wIPV groups compared to one-month post-booster immunization were 10.88 vs. 13.33, 8.08 vs. 7.10, and 7.39 vs. 7.40, respectively. At the age of 6.5 years, the reductions were 19.35 vs. 23.42, 28.12 vs. 23.83, and 32.45 vs. 34.54, respectively. The reduction folds were essentially similar between the two groups, with only the serotype I showing a statistically significant difference (*p* = 0.0343).

Further analysis of the immunogenicity at this observation point showed that the difference in nAbs SPRs (95% CI) were 0.76 (−0.74, 4.17), 0.73 (−1.55, 4.63), and 0.76 (−0.74, 4.17), with the lower limit of the 95% CI being greater than −10%, indicating non-inferiority; the ratio of GMTs for each serotype nAbs (95% CI) were 3.02 (2.46, 3.71), 2.28 (1.87, 2.78), and 1.92 (1.52, 2.42), with the lower limit of the 95% CI being greater than 0.67, indicating non-inferiority.

**Figure 2:**
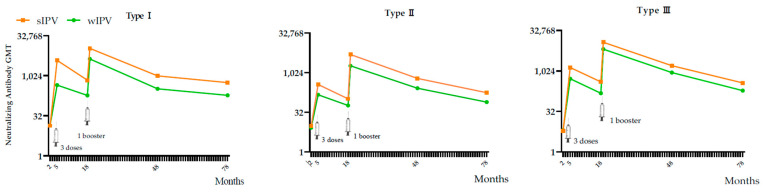
nAb GMT at different observation points from baseline to 5 years after the fourth dose (age 6.5 years) in the IPS3.

The change in nAbs levels from the baseline (2 months of age) before the primary immunization in Phase III to 5 years after the completion of the four-dose immunization regimen (age 6.5 years) is shown in Figure 2.

## 4. Discussion

This study offers comprehensive data on the immune persistence of the novel Sinovac sIPV vaccine, encompassing data from 2.5 years to 5 years post-vaccination with four doses. Across various observation time points, ranging from one month post-primary vaccination to the fifth year following the fourth dose, the nAb GMTs for serotypes I–III in the sIPV group were significantly higher compared to the wIPV group (*p* < 0.0001). Concurrently, the sIPV groups exhibited non-inferior immune responses when compared with the wIPV control group, as evidenced by comparable SPRs and GMTs at 2.5 years and 5 years post-administration of the four-dose regimen.

After receiving four doses, the SPRs for nAbs against serotypes 1–3 in the sIPV group and wIPV group approached 100% at ages 4 and 6.5. Although the strains assayed in this study were of the Sabin type, the completed cross-neutralization tests demonstrated that the Sabin strain vaccine exhibited good cross-neutralization capabilities against the Salk strain, which is the antigen type contained in the wIPV [14]. Meanwhile, the comparison between the sIPV and wIPV groups revealed that the fold decreases in GMTs for each serotype one month after the booster immunization were essentially consistent between the two groups, except for serotype I at age 4, which was significantly lower in the sIPV group compared to the wIPV group (*p* = 0.0343).

Currently, data on the immune persistence of stand-alone IPV is relatively scarce, with most studies focusing on combined vaccines [22,23,24,25,26]. Our study is one of the few with the largest sample size regarding the immunogenicity persistence of stand-alone sIPV (including the supplementary analysis of IPS3). A study on the immune persistence following four doses of Sanofi’s quadrivalent vaccine (DTaP-IPV) showed that the nAbs SPRs for serotype 3 (the serotype with the lowest antibody levels in the results) of wIPV was 93.6% at the age of 4 and 94.6% at the age of 6, slightly lower than the results from this study [27]. Another study adhering to the 2010 French immunization schedule (infants must complete three doses of IPV-containing combined vaccines within six months after birth, and receive three boosters at 16–18 months, 6 years, and 11–13 years of age) demonstrated that one month after the Td-IPV booster at 6-year-old (i.e., after the fifth dose), the GMT of nAbs for IPV were 4776.8 (95% CI, 4093.4–5574.2), 5715.4 (4919.4–6640.2), and 6016.0 (5138.4–7043.4) [28]. Due to the unreported antibody levels before the booster dose at age 6, direct comparison is impossible [28]. But before the booster at 11–13 years of age, the nAbs GMT were 233 (186–291), 405 (338–484), and 314 (255–388), which are generally close to the antibody levels observed in this study five years after the fourth dose, i.e., at age 6.5 [29]. The nAb GMT in 6-year-old children following a booster dose with IPV-containing combined vaccines were not only lower than the sIPV antibody levels observed at 6.5 years in this study but also fell below the levels one month post the fourth dose of booster with the same wIPV, closing to the serological surveillance estimates for one month after four doses of wIPV monovalent vaccine in the Hong Kong population: 2068 (95% CI, 1517–2864), 4705 (3439–6436), and 2758 (1894–4086) [11]. This reflects, to some extent, the potential antigenic interference effect of IPV-containing combined vaccines, thereby increasing the necessity for more booster doses [30].

The sIPV vaccine, developed by the Medical Biology Institute of the Chinese Academy of Medical Sciences (Kunming Institute), has been on the market since 2015. Its immune persistence study over 8.5 years [31] shows that among participants who completed the 4-dose vaccination, the GMT of serotypes 1–3 antibodies for the sIPV and wIPV groups at the age of 4 were 2593.4 vs. 692.1, 645.1 vs. 356.7, and 672.0 vs. 549.7, respectively. Compared to 30 days after the booster, the fold decrease in nAb for each serotype was 5.1 vs. 5.7, 12.2 vs. 8.0, and 9.6 vs. 9.1. At the age of 6, the GMT of serotype 1–3 antibodies for the sIPV and wIPV groups were 2398.5 vs. 649.3, 477.3 vs. 209.1, and 507.4 vs. 300.3, respectively, with a fold decrease of 5.5 vs. 6.0, 16.5 vs. 13.6, and 12.7 vs. 16.6 compared to one month after the booster. Due to differences in antigen content among vaccines from different manufacturers and detection errors, direct comparison of study results may be biased, especially in the interpretation of dilution titers in neutralization tests where a variation of one or two dilution titers is quite normal, direct comparison of neutralizing antibody titer results may yield biases. Therefore, caution should be exercised when analyzing and interpreting neutralizing antibody titer data across different studies. However, the results above, consistent with those of this study, show that at around the age of 6, the nAbs of sIPV remain at a relatively high level. In this study, the fold decrease in nAbs for the sIPV group compared to the wIPV group was essentially the same, and at 6.5 years of age, the immunogenicity of the sIPV group was non-inferior to the wIPV group, with nAbs SPR approaching 100.00%, indicating that the vaccine can provide substantial protection effect for children aged 6.5 years in practical application.

Furthermore, considering that 90% of poliomyelitis cases occur in children under the age of 5, the seroprevalence study conducted in Hong Kong among populations vaccinated with four or five doses of wIPV estimates that after four doses of IPV, 90% of children maintain a serum nAb level of at least 1:8 for at least 7 years; it is hypothesized that after five doses of IPV, the waning rates of antibody decline is slower than in those immunized with four doses, with expected maintenance of at least 10 years [11]. However, model-based analyses struggle to accurately simulate real-world immune responses within populations, hence the necessity for long-term follow-up studies on children vaccinated with four doses of sIPV to more precisely evaluate the nAb against poliovirus. Consequently, we will conduct a 10-year follow-up study on the immune persistence of participants who have received four doses of the sIPV vaccine in Phase III clinical trial.

Our study also has some limitations. Firstly, experts posit that sustained antibody levels are widely recognized as a valid metric for gauging the durability of vaccine-induced immunity. Nonetheless, immune memory serves as a valuable adjunct to immune persistence. This immune memory response can endure for extended periods even after specific nAb levels have waned. Upon re-exposure to the pathogen, it can swiftly mobilize a protective response [32]. However, this study did not encompass the detection and assessment of immune memory and the associated immune memory cells.

Considering the pivotal role of adults and adolescents in disease transmission, as well as the potential increase in exposure risk to cVDPV following waning immunity in adolescents or adults, future seroprevalence studies among a broader age demographic, and evaluations of seroprevalence studies following the introduction of new vaccination programs in high-risk areas, will provide additional scientific evidence for the global goal of polio eradication and the formulation of immunization strategies [1,11].

Ultimately, although combined vaccines have the advantage of reducing the number of doses administered to children at the same time, further attention must be paid to the immunogenicity and immune persistence of the components in combined vaccines when developing combined immunization or co-administration in the future.

## 5. Conclusions

Sinovac’s sIPV has shown remarkable immune persistence, with a vaccination schedule of one dose at 2, 3, 4 months, and 18 months. The study indicates that the nAb SPRs (≥8) for each serotype in participants is nearly 100% at ages 4 and 6.5 years. Moreover, the nAb SPRs and GMT of each serotype in the sIPV group are non-inferior to those in the wIPV group, suggesting that immune persistence remains robust five years post-sIPV administration, with antibody levels well above the internationally recognized threshold for protection against poliovirus. Consequently, no booster immunization is indicated at 6.5 years.

## Figures and Tables

**Table 1:** Participant demographics and other baseline information (IPS2, IPS3).

Immune Persistence Set	Characteristics	sIPV Group	wIPV Group	*p*-Value
IPS2	N	318	165	
	Age (Months), mean ± (SD)	80.0 (0.82)	80.0 (0.75)	0.6784
	Male *n* (%)	180 (56.60)	84 (50.91)	0.2332
	Female *n* (%)	138 (43.40)	81 (49.09)	
IPS3	N	255	132	
	Age (Months), mean ± (SD)	79.9 (0.82)	80.0 (0.76)	0.9925
	Male *n* (%)	144 (56.47)	67 (50.76)	0.2846
	Female *n* (%)	111 (43.53)	65 (49.24)	

**Table 2:** nAb SPRs (≥1:8) and GMTs for serotypes I–III following the fourth dose of vaccination at 5 years (IPS2).

Serotype	Variable	sIPV Group	wIPV Group	*p*-Value	SPRs Differences or GMT Ratios	Non-Inferiority Test (*p*-Value)
1	N	318	165			
	SPRs (95% CI)	99.69 (98.26,99.99)	99.39 (96.67,99.98)	1.0000	0.29	1.0000
	GMTs (95% CI)	543.96 (483.85,611.55)	190.75 (163.58,222.43)	<0.0001	2.85	<0.0001
2	N	334	166			
	SPRs (95% CI)	99.37 (97.75,99.92)	98.79 (95.69,99.85)	0.6089	0.58	0.6089
	GMTs (95% CI)	179.59 (160.80,200.58)	81.05 (70.66,92.98)	<0.0001	2.22	<0.0001
3	N	334	166			
	SPRs (95% CI)	100.00 (98.85,100.00)	99.39 (96.67,99.98)	0.3416	0.61	0.3416
	GMTs (95% CI)	362.72 (328.43,400.58)	203.95 (169.47,245.45)	<0.0001	1.78	<0.0001

**Table 3:** nAb GMT and SPR at different observation points from baseline to 5 years after the fourth dose (age 6.5 years) in the IPS3.

Variable	SPR			GMT		
	sIPV Group (*n* = 255)	IPV Group (*n* = 132)	*p*-Value	sIPV Group (*n* = 255)	IPV Group (*n* = 132)	*p*-Value
Serotype 1						
2M	60.78 (54.50,66.82)	61.36 (52.50,69.71)	1.0000	13.62 (11.61,15.97)	13.83 (11.12,17.20)	0.9101
5M	100.00 (98.56,100.00)	100.00 (97.24,100.00)	NA	3884.34 (3440.83,4385.01)	456.24 (393.93,528.41)	<0.0001
18M	99.61 (97.83,99.99)	100.00 (97.24,100.00)	1.0000	696.04 (600.40,806.91)	186.10 (152.58,226.97)	<0.0001
19M	100.00 (98.56,100.00)	100.00 (97.24,100.00)	NA	10,954.29 (10230.07,11729.78)	4389.82 (3854.88,4998.99)	<0.0001
48M	100.00 (98.56,100.00)	100.00 (97.24,100.00)	NA	1006.42 (884.28,1145.44)	329.30 (278.62,389.19)	<0.0001
19M/48M				10.88 (9.78,12.12)	13.33 (11.37,15.63)	0.0343
78M	100.00 (98.56,100.00)	99.24 (95.85,99.98)	0.3411	566.01 (499.92,640.84)	187.41 (158.73,221.27)	<0.0001
19M/78M				19.35 (17.42,21.50)	23.42 (20.09,27.32)	0.0408
Serotype 2						
2M	51.76 (45.45,58.04)	42.42 (33.87,51.32)	0.0869	9.73 (8.55,11.06)	8.35 (6.92,10.07)	0.1782
5M	100.00 (98.56,100.00)	100.00 (97.24,100.00)	NA	363.88 (324.35,408.23)	147.38 (125.13,173.60)	<0.0001
18M	97.65 (94.95,99.13)	96.97 (92.42,99.17)	0.7403	103.21 (89.75,118.68)	58.08 (47.52,71.00)	<0.0001
19M	100.00 (98.56,100.00)	100.00 (97.24,100.00)	NA	4992.32 (4504.67,5532.76)	1858.72 (1611.97,2143.24)	<0.0001
48M	99.61 (97.83,99.99)	100.00 (97.24,100.00)	1.0000	617.83 (537.32,710.40)	261.70 (223.70,306.15)	<0.0001
19M/48M				8.08 (7.20,9.07)	7.10 (6.11,8.26)	0.1892
78M	99.22 (97.20,99.90)	98.48 (94.63,99.82)	0.6082	177.55 (156.63,201.27)	78.01 (66.78,91.14)	<0.0001
19M/78M				28.12 (25.31,31.23)	23.83 (20.50,27.70)	0.0732
Serotype 3						
2M	25.88 (20.62,31.72)	25.00 (17.88,33.28)	0.9025	5.99 (5.41,6.62)	6.40 (5.45,7.51)	0.4723
5M	100.00 (98.56,100.00	100.00 (97.24,100.00)	NA	1364.22 (1226.61,1517.28)	523.40 (443.43,617.79)	<0.0001
18M	100.00 (98.56,100.00)	97.73 (93.50,99.53)	0.0391	399.13 (347.01,459.09)	151.03 (115.27,197.90)	<0.0001
19M	100.00 (98.56,100.010)	100.00 (97.24,100.00)	NA	11,860.65 (11215.30,12543.14)	6573.40 (5632.16,7671.93)	<0.0001
48M	100.00 (98.56,100.00)	100.00 (97.24,100.00)	NA	1604.49 (1425.47,1806.01)	888.74 (725.34,1088.97)	<0.0001
19M/48M				7.39 (326.79,408.73)	7.40 (155.25,233.30)	0.9959
78M	100.00 (98.56,100.00)	99.24 (95.85,99.98)	0.3411	365.47 (326.79,408.73)	190.31 (155.25,233.30)	<0.0001
19M/78M				32.45 (29.22,36.04)	34.54 (29.26,40.77)	0.5140

2M, Baseline; 5M, 1-month post-primary immunization; 18M, before booster immunization; 19M, 1-month post-booster immunization; 48M, 2.5 years post-booster immunization (age 4 years); 78M, 5 years post-booster immunization (age 6.5 years).

## Data Availability

Data are contained within the article.
